# FKBP12 binds to the cardiac ryanodine receptor with negative cooperativity: implications for heart muscle physiology in health and disease

**DOI:** 10.1098/rstb.2022.0169

**Published:** 2023-06-19

**Authors:** S. J. Richardson, C. G. Thekkedam, M. G. Casarotto, N. A. Beard, A. F. Dulhunty

**Affiliations:** John Curtin School of Medical Research, Australian National University, Canberra, Australia, Australian Capital Territory 2601, Australia

**Keywords:** ryanodine receptor, FKBP12, FKBP12.6, sarcoplasmic reticulum, Ca^2+^ release

## Abstract

Cardiac ryanodine receptors (RyR2) release the Ca^2+^ from intracellular stores that is essential for cardiac myocyte contraction. The ion channel opening is tightly regulated by intracellular factors, including the FK506 binding proteins, FKBP12 and FKBP12.6. The impact of these proteins on RyR2 activity and cardiac contraction is debated, with often apparently contradictory experimental results, particularly for FKBP12. The isoform that regulates RyR2 has generally been considered to be FKBP12.6, despite the fact that FKBP12 is the major isoform associated with RyR2 in some species and is bound in similar proportions to FKBP12.6 in others, including sheep and humans. Here, we show time- and concentration-dependent effects of adding FKBP12 to RyR2 channels that were partly depleted of FKBP12/12.6 during isolation. The added FKBP12 displaced most remaining endogenous FKBP12/12.6. The results suggest that FKBP12 activates RyR2 with high affinity and inhibits RyR2 with lower affinity, consistent with a model of negative cooperativity in FKBP12 binding to each of the four subunits in the RyR tetramer. The easy dissociation of some FKBP12/12.6 could dynamically alter RyR2 activity in response to changes in *in vivo* regulatory factors, indicating a significant role for FKBP12/12.6 in Ca^2+^ signalling and cardiac function in healthy and diseased hearts.

This article is part of the theme issue ‘The heartbeat: its molecular basis and physiological mechanisms’.

## 1. Introduction

The ryanodine receptor Ca^2+^ release channel (RyR) is embedded in the sarcoplasmic reticulum (SR) Ca^2+^ store membrane of striated muscle and releases the stored Ca^2+^ to enable contraction. There are three RyR isoforms expressed in many different tissues. RyR1 is the major isoform expressed in skeletal muscle and RyR2 in cardiac muscle. RyR channel opening and Ca^2+^ release from the SR *in vivo* depend on intrinsic channel gating mechanisms and on soluble factors that bind to the protein. These regulatory factors include the 12.0 and 12.6 kDa FK506 binding proteins, FKBP12 and FKBP12.6. There is one FKBP binding site for both isoforms on each of the four subunits of the RyR tetramer [1], which were visualized in early cryo-electron microscopy (CryoEM) studies [2] and later in high-resolution CryoEM structures [1,3]. Often fatal cardiac arrhythmias and skeletal myopathies are associated with the dissociation of these proteins from the RyRs. FKBP12 and FKBP12.6 are expressed in heart [4] but bind to RyR2 in species-specific ratios [5,6], with different ratios in mice with different genetic backgrounds [7]. In 57BL6/J mice, FKBP12 binding to RyR2 is approximately 100-fold greater than FKBP12.6 [6]. Both isoforms bind to RyR2 from sheep and humans, with FKBP12 favoured over FKBP12.6 [8,9].

Although the intimate association between FKBPs and RyRs is well recognized, the functional effects of the FKBPs binding to RyR2 are disputed. Reported influences of FKBP on isolated RyR2 channels vary from no effect of either isoform on canine or mouse RyR2, or on RyR2 co-expressed with FKBP12.6 [10–12], to FKBP12 activating sheep heart RyR2 while FKBP12.6 has no effect, although it prevents activation by FKBP12 [13]. The impact of RyR2 phosphorylation is also debated as FKBP12.6 inhibits PKA phosphorylated RyR2 in some reports [14,15] but does not in other studies [7]. Species differences likely contribute to varying observations, as well as the methods used to strip the FKBPs from RyRs [8]. In whole-cell studies, the parameters measured and the preparation examined may be additional variables. For example, FKBP12.6 did not influence susceptibility to stress-induced arrhythmia in rat myocytes [12], but FKBP12 and FKBP12.6 reduced spontaneous Ca^2+^ release in HEK cells, indicating a critical inhibitory regulation of RyR2 [16].

Here, we focus firstly on the functional effects of FKBP12 binding to RyR2, which, except in Galfré *et al*. [13], have not been addressed in detail. This is a physiologically significant question given that FKBP12 is a major isoform associated with RyR2 in many species, including humans. We find significant effects of FKBP12 on RyR2 activity, with activation followed by inhibition as FKBP exposure time and concentration increases. Co-immunoprecipitation (CoIP) methods were employed to examine FKBP12 association with RyR2, revealing that a proportion of FKBP is bound tightly to the RyR2 tetramers while a fraction is easily dissociated. Since each monomer of the RyR2 tetramer contains a binding site for FKBP, we suggest a model in which channel activity increases when FKBP12 binds to one or two of the subunits and then declines as more subunits become FKBP12-bound. We further suggest that the results are consistent with negative cooperativity in FKBP binding to RyR2. Overall, the results have significant implications for the cellular impact of FKBP12 on cardiac function.

## 2. Methods

Details of individual procedures are given in the electronic supplementary material, Methods.

### (a) Preparation of sheep cardiac sarcoplasmic reticulum vesicles and RyR2 solubilization

**Figure 1.**
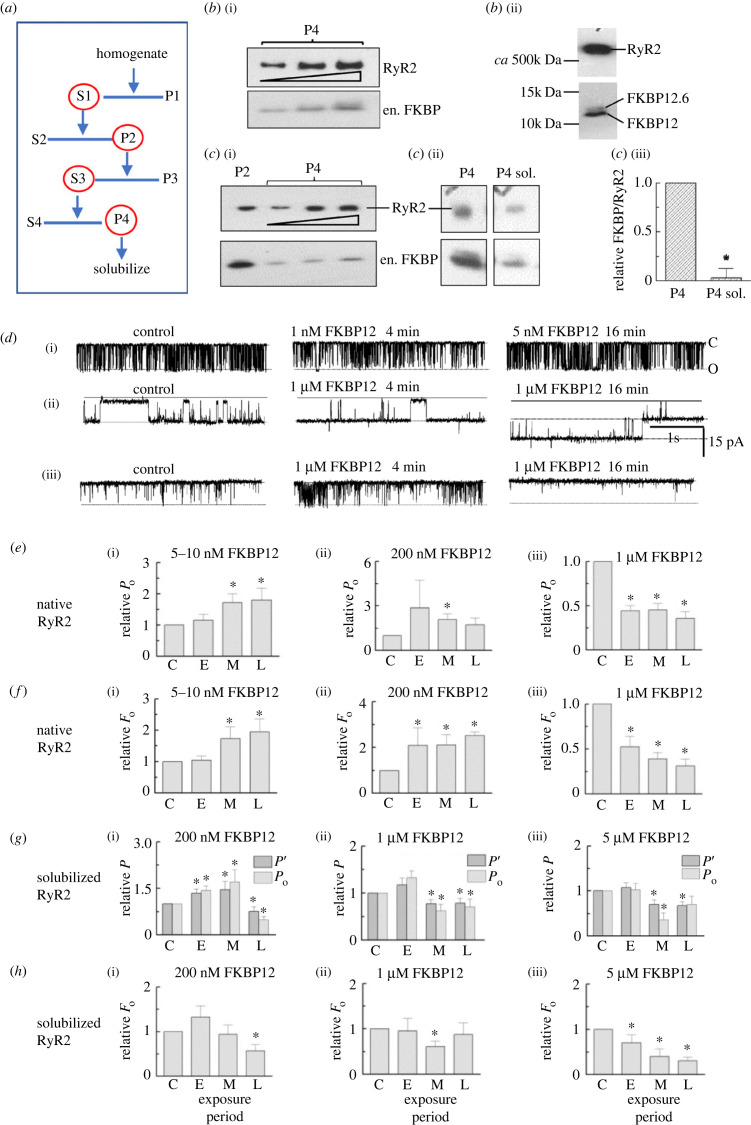
Isolation and characterization of RyR2 channels from sheep cardiac sarcoplasmic reticulum (SR) vesicles and solubilized SR. (*a*–*c*) General considerations. Sequential centrifugation steps in isolating SR vesicles enriched in RyR2 channels and solubilized RyR2. Solute fractions are labelled S1–S4 and precipitate fractions labelled P1–P4. (*b*) (i) Western blot showing approximate linearity in RyR2 and FKBP12/12.6 band densities following co-immunoprecipitation (CoIP) of RyR2 from P4 vesicles, loaded at protein concentrations of approximately 3, 6 and 9 µg (left to right) (electronic supplementary material, figure S5). (ii) Blots from one lane indicating FKBP12.6 and FKBP12 and positions of molecular mass markers around the FKBPs and approximate position of 500 kDa. (*c*) (i) A preliminary CoIP experiment suggesting FKBP associated with RyR2 is less in P4 vesicles than in P2 vesicles (electronic supplementary material, figure S5). (ii) Less FKBP is associated with RyR2 following CoIP of solubilized protein, compared with that of P4 vesicles. (iii) The ratio of densities of FKBP12/12.6 bands to RyR2 bands (FKBP/RyR2) in P4 and solubilized fractions, normalized to FKBP/RyR2 in P4 (*n* = 3). The *n* value refers to the number of individual blots. The ratios were first calculated for RyR and FKBP bands in the same lanes and then expressed relative to ratios for control lanes in the same blot (electronic supplementary material, figure S6). Original Blots for (*b*) and (*c*) are shown in electronic supplementary material, figures S5 and S6. (*d*) Current recordings obtained during typical experiments with FKBP12 addition to the cytoplasmic solution reveal a biphasic response in solubilized RyR2 channels. Recordings from three experiments (i,ii*,*iii) before FKBP12 addition (control) and at indicated [FKBP12] and times after adding FKBP12. Currents at −40 mV, with downward deflections indicating channel opening. The examples show no change in activity (i), increased activity (ii) or increased activity followed by decreased activity (iii). Each panel is from the same channel before (control) and 4 min, then 16 min, after FKBP12 application. (*e*–*h*) Average time- and concentration- dependent changes in RyR2 activity resulting from cytoplasmic addition of FKBP12. Channels were from P4 vesicles (*e*,*f*) and solubilized RyR2 (*g*,*h*). Average relative open probability (*P*_o_, or *P'* defined in the text) plotted in (*e*) and (*g*), and average relative frequency of opening (*F*_o_) plotted in (*f*) and (*h*). Average parameter values (*P*_o_, *T*_o_, *T*_c_ and *F*_o_) are given in tables 1 and 2, along with numbers of observations and average values for all measured parameters. [FKBP12] is shown with each graph from lowest (left column), to intermediate (central column) and highest (right column). Data were obtained under control condition (C) and then at early (E), intermediate (M) and late (L) times after FKBP12 addition. Times for each data set are listed in tables 1 and 2. The vertical bars show the mean ± s.e.m. Asterisks indicate significant changes after the addition of FKBP12. (Online version in colour.)

SR vesicles were prepared [17] from sheep heart, and RyR2 was solubilized from P4 vesicles (figure 1*a*).

**Table 1.** Actual and relative parameter values for native RyR2 at indicated [FKBP12] and times after FKBP12 addition. Red font—significant changes indicating RyR2 activation. Blue font—significant changes indicating of RyR2 inhibition. *n* is the number of observations; *n* s^−1^ is the number of open events per second. Times given for 5–10 nM FKBP12 are the times after the first addition of 5 nM FKBP12. Average relative values were calculated for individual experiments before determination of the mean ± s.e.m (see Methods and electronic supplementary material, methods).

		control, mean ± s.e.m.	early, mean ± s.e.m.	intermediate, mean ± s.e.m.	late, mean ± s.e.m.
			time (min) after FKBP12 addition		
**5–10 nM** **FKBP12**			6–12	13–18	19–28
	*P*_o_	0.0104 ± 0.0035	0.0114 ± 0.0042	0.01433 ± 0.0040	0.0151 ± 0.0029
*n* = 10	relative *P*_o_	1	1.152 ± 0.191	1.721 ± 0.274	1.803 ± 0.337
	*T*_o_ (ms)	1.329 ± 0.169	1.375 ± 0.202	1.373 ± 0.23	1.258 ± 0.267
	relative *T*_o_	1	0.118 ± 0.13	1.063 ± 0.118	1.144 ± 0.280
	*T*_c_ (ms)	273.61 ± 63.95	251.10 ± 73.64	256.82 ± 83.00	199.48 ± 67.79
	relative *T*_c_	1	1.0557 ± 0.0791	0.9118 ± 0.2192	0.7110 ± 0989
	*F*_o_ (*n* s^−1^)	7.258 ± 1.711	8.7912 ± 2.1123	12.7210 ± 3.8497	11.7585 ± 2.446
	relative *F*_o_	1	1.0483 ± 0.1379	1.7279 ± 0.3747	1.9435 ± 0.4153
				
**200 nM** **FKBP12**			1–3	5–11	13–23
*n* = 6	*P*_o_	0.0061 ± 0.0023	0.0320 ± 0.0191	0.0134 ± 0.0077	0.0019 ± 0.0007
	relative *P*_o_	1	2.8627 ± 1.8782	2.0911 ± 0.4054	1.7413 ± 0.4805
	*T*_o_ (ms)	0.74 ± 0.1416	2.198 ± 1.025	0.923 ± 0.177	0.493 ± 0.045
	relative *T*_o_	1	4.108 ± 2.432	1.694 ± 0.694	0.772 ± 0.221
	*T*_c_ (ms)	701.49 ± 305.58	380.78 ± 135.42	327.88 ± 121.34	487.13 ± 204.05
	relative *T*_c_	1	1.258 ± 0.560	1.340 ± 0.881	0.0447 ± 0.0523
	*F*_o_ (*n* s^−1^)	13.83 ± 10.48	7.4936 ± 3.0859	10.5517 ± 5.3393	3.7469 ± 1.5561
	relative *F*_o_	1	2.0827 ± 0.7742	2.1025 ± 0.4618	2.5113 ± 0.1568
				
**1 µM** **FKBP12**			1–3	7–10	18–25
*n* = 10	*P*_o_	0.0232 ± 0.0168	0.0032 ± 0.0014	0.0035 ± 0.0018	0.0028 ± 0.0017
	relative *P*_o_	1	0.4423 ± 0.06	0.4452 ± 0.0743	0.3572 ± 0.0788
	*T*_o_ (ms)	0.797 ± 0.148	0.737 ± 0.165	0.890 ± 0.154	0.922 ± 0.2042
	relative *T*_o_	1	0.924 ± 0.081	1.1953 ± 0.1219	1.1626 ± 0.1064
	*T*_c_ (ms)	260.35 ± 71.717	434.717 ± 96.5377	606.743 ± 133.909	789.608 ± 195.341
	relative *T*_c_	1	3.9767 ± 1.4468	4.2377 ± 1.3742	6.6911 ± 2.1824
	*F*_o_ (*n* s^−1^)	21.43 ± 13.21	3.6483 ± 0.8868	3.6294 ± 1.2656	2.1964 ± 0.6954
	relative *F*_o_	1	0.5264 ± 0.1157	0.3902 ± 0.0717	0.3132 ± 0.0752
				
**5 µM** **FKBP12**			2	9	19
*n* = 2	*P*_o_	0.0014	0.0018	0.00075	0.0006
	relative *P*_o_	1	1.2216	0.5114	0.4176
	*T*_o_ (ms)	0.61	0.65	0.515	0.54
	relative *T*_o_	1	1.0117	0.844	0.881
	*T*_c_ (ms)	446.55	396.20	845.37	997.03
	relative *T*_c_	1	0.8989	1.9588	2.2886
	*F*_o_ (*n* s^−1^)	2.25	2.53	1.33	1.05
	relative *F*_o_	1	1.1398	0.6099	0.4785

**Table 2.** Actual and relative parameter values for solubilized RyR2 at indicated [FKBP12] and times after FKBP12 addition. Red font - significant changes indicative of RyR2 activation. Blue font - significant changes indicative of RyR2 inhibition. *n* is the number of observations; n s^−1^ is the number of open events per second. Average relative values were calculated for individual experiments before determination of the mean ± s.e.m (see Methods and electronic supplementary material, methods). *P'* is the average combined probability of opening in single and multiple channel recordings, as defined in the Results and electronic supplementary material, methods.

		control, mean ± s.e.m.	early, mean ± s.e.m.	intermediate, mean ± s.e.m.	late, mean ± s.e.m.
			time (min) after adding FKBP12		
**200 nM** **FKBP12**			1–3	6–12	14–22
*n* = 10	*P′*	0.05858 ± 0.0254	0.0881 ± 0.0392	0.0864 ± 0.3384	0.0517 ± 0.028
	relative *P′*	1	1.3401 ± 0.136	1.4578 ± 0.2591	0.7571 ± 0.1442
*n* = 6	*P*_o_	0.02848 ± 0.0113	0.04386 ± 0.0198	0.0552 ± 0.026	0.0019 ± 0.0007
	relative *P*_o_	1	1.4442 ± 0.1490	1.7120 ± 0.3893	0.48139 ± 0.1149
	*T*_o_ (ms)	1.022 ± 0.241	1.052 ± 0.232	1.105 ± 0.0.232	0.635 ± 0.037
	relative *T*_o_	1	1.046 ± 0.071	1.118 ± 0.114	0.934 ± 0.107
	*T*_c_ (ms)	44.45 ± 10.53	38.14 ± 9.65	74.62 ± 28.27	115.92 ± 29.47
	relative *T*_c_	1	0.904 ± 0.085	1.464 ± 0.325	2.599 ± 0.679
	*F*_o_ (*n* s^−1^)	37.584 ± 17.22	35.072 ± 8.886	32.783 ± 12.310	11.464 ± 2.249
	relative *F*_o_	1	1.3262 ± 0.2538	0.9383 ± 0.2100	0.5689 ± 0.1414
				
**1 µM** **FKBP12**			1–3	6–10	12–22
*n* = 14	*P′*	0.3931 ± 0.0665	0.4129 ± 0.0737	0.3346 ± 0.0580	0.3425 ± 0.0634
	relative *P′*	1	1.1702 ± 0.154	0.7745 ± 0.0878	0.7846 ± 0.1010
*n* = 6	*P*_o_	0.3643 ± 0.1361	0.3998 ± 0.1493	0.2687 ± 0.00972	0.3470 ± 0.1369
	relative *P*_o_	1	1.3315 ± 0.2862	0.6220 ± 0.1400	0.7073 ± 0.1678
	*T*_o_ (ms)	3.0866 ± 1.7347	2.5220 ± 1.44	3.525 ± 2.3283	2.0383 ± 0.8570
	relative *T*_o_	1	1.8348 ± 0.9371	0.9587 ± 0.0903	0.8073 ± 0.0758
	*T*_c_ (ms)	11.2183 ± 7.2330	19.258 ± 10.793	47.693 ± 37.714	44.5167 ± 34.25
	relative *T*_c_	1	11.765 ± 10.848	3.9769 ± 1.7158	2.0712 ± 0.6486
	*F*_o_ (*n* s^−1^)	149.173 ± 43.343	120.187 ± 34.408	117.230 ± 46.447	148.013 ± 49.977
	relative *F*_o_	1	0.9553 ± 0.2713	0.6145 ± 0.1200	0.8814 ± 0.2453
				
**5 µM** **FKBP12**			1–3	6–12	15–23
*n* = 18	*P′*	0.3638 ± 0.0498	0.3992 ± 0.0658	0.3128 ± 0.0587	0.2624 ± 0.0484
	relative *P′*	1	1.0718 ± 0.1078	0.7074 ± 0.0972	0.6792 ± 0.0819
*n* = 4	*P*_o_	0.1039 ± 0.0766	0.1500 ± 0.1178	0.0723 ± 0.0648	0.01111 ± 0.0832
	relative *P*_o_	1	1.0206 ± 0.2638	0.3634 ± 0.1560	0.7416 ± 0.1809
	*T*_o_ (ms)	1.0650 ± 0.34603	1.7400 ± 0.6996	0.8775 ± 0.2520	4.6875 ± 3.2652
	relative *T*_o_	1	1.4727 ± 0.2058	0.9160 ± 0.1570	3.2820 ± 1.6193
	*T*_c_ (ms)	103.495 ± 53.560	267.632 ± 199.104	599.862 ± 347.027	392.028 ± 186.230
	relative *T*_c_	1	1.9632 ± 0.8889	3.9572 ± 1.2462	4.5919 ± 1.7922
	*F*_o_ (*n* s^−1^)	67.513 ± 49.317	51.838 ± 39.083	51.658 ± 45.072	29.9041 ± 27.087
	relative *F*_o_	1	0.7029 ± 0.1813	0.4042 ± 0.1661	0.3085 ± 0.0864

### (b) Lipid bilayers and RyR2 channel incorporation

Lipid bilayers were formed from a mixture of phosphatidylethanolamine, phosphatidylserine and phosphatidylcholine (in n-decane) as previously described [18,19]. The SR vesicles were added to the *cis* solution and incorporated into bilayers with their cytoplasmic surface facing the *cis* chamber. RyR2 channel activity was recorded with symmetrical 250 mM Cs^+^ in the *cis* and *trans* chambers, with physiological diastolic Ca^2+^ concentrations of 1 µM Ca^2+^ in the *cis* chamber and 1 mM Ca^2+^ in the *trans* chamber.

### (c) Single channel recording and analysis

Electrodes in the *cis* and *trans* solutions detected current flow through RyR2 channels and voltage clamped the potential across the bilayer (*V*_*cis*_ − *V*_*trans*_), which was switched between −40, 0 and +40 mV every 30 s throughout the experiment. Channel activity was analysed over consecutive periods of 60–90 s at each potential, under each condition. When one channel only was active in a bilayer, open probability (*P*_o_), mean open time (*T*_o_), and mean closed time (*T*_c_) were measured using the in-house programs Channel 2 or Channel 3, with open channel discriminators set above the baseline noise at approximately 20% of the maximum single channel conductance. When two to four channels were active, *P*_o_ was approximated from the mean current, *I'* (Results; electronic supplementary material, methods and figures S1–S4) [19]. Channel activity was analysed before FKBP12 addition, then after adding FKBP12, at early, intermediate, and later times (up to 30 min) post addition. Specific times are given in the Results.

Because RyR activity characteristically varies between channels [20], parameter values during exposure to FKBP12 were expressed relative to the internal control values for individual channels to give equal weighting to the effect of FKBP12 on all channels [19]. In other words, the parameter values determined for each bilayer were normalized to the internal control value obtained for that bilayer before FKBP12 addition. The average relative data presented in the manuscript are the means of the relative values calculated for each individual bilayer experiment. This is addressed in more detail in electronic supplementary material, figures S3 and S4. The analyses of all ion channel recordings are given in the supplementary material, channel data files.

### (d) FKBP12 expression and purification

GST-FKBP12 was expressed in *Escherichia coli* in a pGEX2TK vector and purified by glutathione (GST)–agarose affinity chromatography [21]. GST-FKBP12 or GST-free FKBP12 (following thrombin cleavage) were purified by fast protein liquid chromatography (FPLC) [8].

### (e) SDS-PAGE and Western blotting

Vesicle proteins were separated by SDS-PAGE, and protein bands were stained with Coomassie blue or transferred to polyvinylidene difluoride (PVDF) membrane. Non-specific antibody binding was blocked with skimmed milk powder or bovine serum albumin (BSA) in phosphate-buffered saline (PBS) containing (mM): 137 NaCl; 7 Na_2_HPO_4_; 2.5 NaH_2_PO_4_.H2O; and 2 EGTA (ethylene glycol-bis[*b*-aminoethyl ether]*N*,*N*,*N*',*N*'-tetraacetic acid), pH adjusted to 7.4. The anti-RyR antibody was 34C (RyR1/RyR2 antibody, Development Studies Hybridoma Bank, USA) diluted 1:6000 in TPBS (0.05% Tween-20 PBS). The FKBP antibody H5 (α-FKBP12/FKBP12.6 antibody, Santa Cruz Biotechnology, USA) detects FKBP12 and FKBP12.6 and was diluted 1 : 1000 in TPBS. The second antibody was 1 : 6000 goat anti-mouse IgG-conjugated HRP [8].

### (f) Co-immunoprecipitation and densitometry

**Figure 2.**
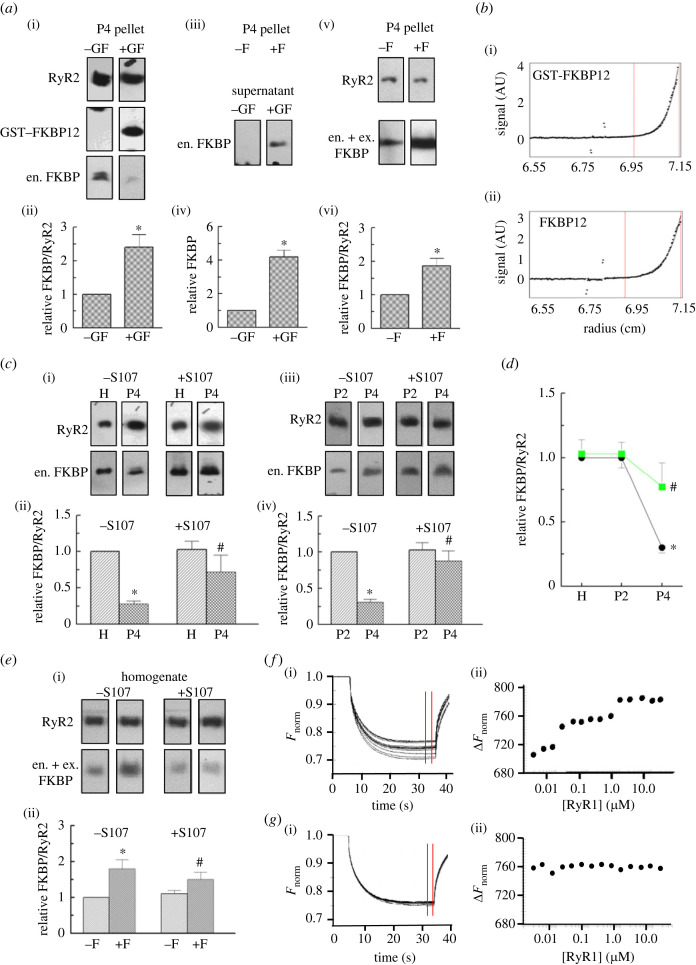
CoIP and Western blot analysis of FKBP12/12.6 bound to RyR2. (*a*) Exogenous (ex.) FKBP12 binding to RyR2 and exchange with endogenous (en.) FKBP12/12.6. (i) P4 vesicles incubated without (−GF) and with (+GF) 10 µM GST-FKBP12 before CoIP (electronic supplementary material, figure S6). (ii) The average of FKBP/RyR2 ratio relative to the −GF ratio (*n* = 4). For +GF, the GST-FKBP12 measure was first divided by 2 to correct for dimerization (see Methods and Results). The resulting ex. FKBP12 number was then added to en. FKBP12/12.6 to obtain the total FKBP (en. + ex. FKBP), which was then divided by the RyR2 density for that lane. The (en. + ex. FKBP)/RyR2 ratio for +BP was then normalized to the control (en. FKBP)/RyR2 ratio in the –BP lane on the same blot. (iii) eFKBP12/12.6 in –GF and +GF supernatants from the experiment in (i) and (ii) (electronic supplementary material, figure S6). (iv) Average en. FKBP12/12.6 in the supernatant normalized to the GF band (*n* = 4). (v) P4 incubated without (−F), and with (+F) 10 µM FKBP12 (i.e. GST-cleaved) (electronic supplementary material, figure S7). (vi) Average FKBP/RyR2 ratios normalized to the −F ratio (*n* = 3). * indicates significant increases in FKBP after incubation with GST-FKBP12 or cleaved FKBP12. (*b*) Analytical ultracentrifugation reveals GST-FKBP12 dimerization. Particle mass from analysis of sedimentation curves between the red vertical lines. AU, arbitrary units. (i) The GST-FKBP12 mass of 72.4 kDa was close to the predicted dimer mass of 76 kDa. (ii) GST-cleaved FKBP12 mass of 12.8 kDa was close to the predicted monomer mass of 12.1 kDa. (*c*) Endogenous FKBP12/12.6 loss during processing is rescued by S107. (i) RyR2 and en. FKBP12/12.6 in homogenate (H) and P4, without (−S107) and with (+S107) 20 µM S107 (electronic supplementary material, figure S7). (ii) Average FKBP/RyR2 normalized to −S107 FKBP/RyR2 (*n* = 5). (iii) Similar to (i), but comparing P2 and P4 (electronic supplementary material, figure S8). (iv) Similar to (ii) but comparing P2 and P4 (*n* = 6). In (*c*), * indicates significant differences between H and P4, or P2 and P4; **#** indicates significant differences between −S107 and +S107. (*d*) Summary: en. FKBP12/12.6 in H, P2 and P4 normalized to H en. FKBP12/12.6. (*e*) RyR2 in homogenate binds additional FKBP12. (i) RyR2 and FKBP12/12.6 from homogenate incubated without (−F) and with (+F) FKBP12, or without (−S107) and with (+S107) 20 µM S107 (electronic supplementary material, figure S8). (ii) Average FKBP/RyR2 in homogenate normalized to −F FKBP/RyR2 (*n* = 5). In (*a*), (c) and (*e*), *n* refers to number of blots. All ratios were first calculated for RyR and FKBP bands in the same test lane, then normalized to ratios in the control lanes of the same blot shown in electronic supplementary material, figures S6–S8. (*f*) Microscale thermophoresis (MST) of FKBP12 binding to rabbit skeletal muscle RyR1. See also Methods and electronic supplementary material, methods. (i) Normalized fluorescence (*F*_norm_) measured for 40 s in the region of the capillary heated by a laser between 5 and 35 s. The migration of fluorescently tagged molecules causes a drop in *F*_norm_. As fluorescently tagged FKBP binds to RyR1, the RyR1 complex migrates more slowly with a smaller change in *F*_norm_. As more FKBP binds to higher [RyR]s, the change in *F*_norm_ becomes progressively smaller. (ii) Δ*F*_norm_ is the difference between the minimum *F*_norm_ in the absence of RyR1 and *F*_norm_ with increasing [RyR], measured between the vertical red lines. (*g*) FKBP12-saturated RyR1 does not bind additional FKBP12. (i) *F*_norm_ does not change with [RyR]. (ii) Δ*F*_norm_ does not change with increasing [RyR1]. In (*f*(i)) and (*g*(i)), the individual curves are light grey in colour, and overlapping curves are darker grey to black as the number of overlapping curves increases. (Online version in colour.)

SR vesicles were incubated without (control) and with FKBP12 or GST-FKBP12 for 1 h at 37°C, then subjected to anti-RyR CoIP [8], followed by SDS-PAGE and Western blot. Western blots were scanned and band density quantified (Bio-Rad ImageLab) and subtracted from background density. An approximately linear relationship between band density and [RyR2] or [FKBP12] was established for 34C and H5 (figure 1*b*(i),*c*(i); electronic supplementary material, figures S5–S8). Because FKBP12.6 was not defined sufficiently clearly for separate densitometry (figure 1*b*(i),*c*(i,ii), figure 2*a,c*,*e*; electronic supplementary material, figures S5–S8), the total density in the area encompassing both FKBP12 and FKBP12.6 was measured as endogenous FKBP (en. FKBP). Unless stated otherwise, the data for each gel are expressed as ratio of FKBP/RyR2 following FKBP12 addition or further vesicle processing relative to the ratio of FKBP/RyR2 before FKBP12 addition or further vesicle processing. Since the densities are measured on the same gels (see legend to figure 2; electronic supplementary material, methods and figures S5–S8), factors such as image exposure time cancel, as do other factors, including antibody affinity. The data reveal changes that occur owing to FKBP12 addition or further vesicle processing, but the analysis does not reveal absolute amounts of either RyR2 or FKBP12. The densitometric analysis data are given supplementary material, Supplementary Densitometry Data file.

### (g) Analytical ultracentrifugation

Analytical ultracentrifugation (AUC) was used to determine the oligomeric state of FKBP12 and GST-FKBP12 [22]. Experimental cells were set up with seven serial dilutions, beginning with 320 µM GST-FKBP12 or FKBP12 and diluting by half for each subsequent dilution. The experiments were run as sedimentation equilibrium assays, with centrifugation at 3000–12 000 × r.p.m., with equilibrium reached after approximately 14 h. Absorbance measurements were obtained in triplicate at 230 and 280 nm. The data were analysed using the Sedphat software package [22]. The analysis confirmed that GST-FKBP12 was present as a dimer. This was taken into account by dividing densitometry measurements of GST-FKBP12 bands by a factor of 2 prior to obtaining the FKBP/RyR ratios and normalization to control ratio as described in the legend to figure 2 and electronic supplementary material, Methods.

### (h) Microscale thermophoresis

Microscale thermophoresis (MST) was performed as recommended (User Manual, Monolith NT.115, NanoTemper). The technique is based on the principle that molecules move along a temperature gradient and that this movement can be used to determine the kinetics of molecular interactions [8,23]. Solubilized RyR1 was serially diluted to yield 14 concentrations ranging from approximately 20 µM to 2 nM. The samples were mixed by brief vortexing with a fixed concentration of a fluorescently tagged FKBP12 immediately before loading into fine capillary tubes. A defined region of the capillary tubes was heated with a laser and fluorescence in the region monitored. Changes in fluorescence as the fluorescently tagged proteins migrated out of the heated region yielded information on the affinity of RyR1 for FKBP12. For a negative control, solubilized rabbit RyR1 was pre-incubated with 10 µM FKBP12, then diluted to 14 concentrations from 20 µM to 2 nM [8,23].

### (i) Statistics

Data are presented as mean ± s.e.m. and significance evaluated using Student's *t*-test with *n* = 4–18 for channel data (details in tables 1 and 2) or *n* = 3–6 for densitometry (details in legends to figures 1 and 2). The effects of FKBP12 on channel activity were not voltage-dependent, so that measurements at +40 and −40 mV were included in the average data, as the two voltages provide independent measures of the effects of FKBP at each concentration with current flow through the pore in opposite directions [19,20].

**Figure 3.**
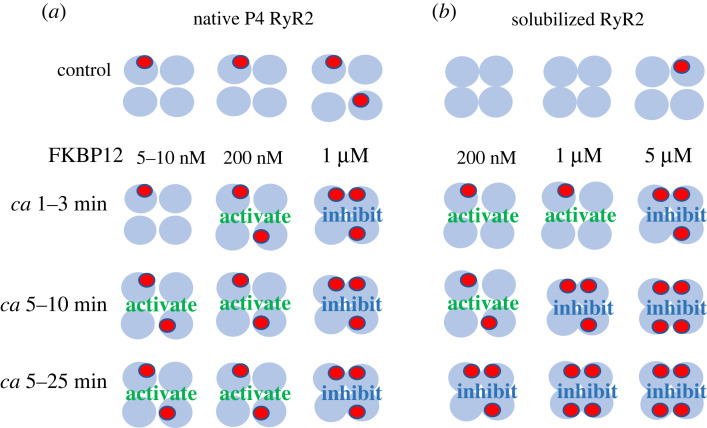
Changes in subunit occupation by FKBP12 within each tetramer might explain the changes in RyR2 channel activity with exogenous FKBP12 binding. (*a,b*) Patterns of FKBP12 occupation of RyR2 subunits in RyR tetramers from P4 vesicles (*a*) and solubilized RyR2 (*b*). The control horizontal panel for P4 shows examples of three control RyR2 tetramers, two with 1 FKBP-bound and one with 2 FKBP-bound molecules, approximating the mean of 1.44 (Results). The control horizontal panel for solubilized RyR2 is shown with two subunits having 0 FKBP-bound, and one RyR2 tetramer with 1 FKBP-bound. This is an overestimate of 1.44 per 10 tetramers, but is shown to indicate a finite number bound rather than 0 (Discussion)*.* The remaining panels show increasing FKBP12 occupation with increasing time after exogenous FKBP12 addition and increasing [FKBP12]. The changes in occupation are suggested to explain the biphasic effects of FKBP12 on RyR2 channel activity presented in figure 1, in the context of data presented in figure 2, as outlined in the Discussion. Note that the activated RyR2 tetramers are illustrated as having a more open conformation, in contrast to the inhibited tetramers, which are depicted in a more closed conformation. (Online version in colour.)

## 3. Results

### (a) Effects of adding exogenous FKBP to RyR2 in lipid bilayers

Native RyR2 in P4 vesicles or solubilized RyR2 (figure 1*a*) was incorporated into lipid bilayers. Native RyR2 remains associated with components of the junctional SR membrane, including triadin, junctin and calsequestrin, which are removed during solubilization [24,25]. Endogenous FKBP12 was reduced during processing to the P4 level and reduced further during solubilization (figure 1*c*(i,ii)). Channel activity was recorded with diastolic physiological levels of 1 µM [Ca^2+^] in the cytoplasmic solution and 1 mM [Ca^2+^] in the luminal solution. GST-cleaved FKBP12 added to the cytoplasmic solution was surprisingly found to increase or decrease solubilized RyR2 channel activity depending on the concentration of FKBP12 and duration of exposure (figure 1*d–h*).

### (b) FKBP12 added to native P4 RyR2

No consistent effect of 1 nM FKBP12 was observed in preliminary experiments (data not shown). Detailed experiments began with 5–10 nM FKBP12, where 5 nM FKBP12 was added initially, and after 10 min a second addition of 5 nM (total 10 nM). The first measurements were after 5 min with 5 nM FKBP12 and later measurements at indicated times after the second 5 nM addition, thus in the presence of 10 nM FKBP12 (table 1). There was no significant change early after 5 nM FKBP12 addition (table 1); however, *P*_o_ increased significantly with 10 nM FKBP12 owing to briefer closures and increased event frequency, but no consistent effect on open times was observed (table 1 and figure 1*e*,*f*). Significant increases in activity were seen at intermediate times with 200 nM FKBP12 (figure 1*e*(ii) and table 1). The FKBP-RyR2 stabilizing compound S107 [26,27] (20 µM) added approximately 10 min after 200 nM FKBP12, did not further alter activity (data not shown). In contrast to lower FKBP12 concentrations, 1–5 µM FKBP12 caused a significant decline in *P*_o__,_ with a significant increase in closed durations and decline in opening frequency (figure 1*e*,*f* and table 1). Average relative *P*_o_ is shown graphically because it reflected overall changes in activity. Relative *F*_o_ is also shown because it most closely reflected changes in *P*_o_, owing to significant changes in the closed times. Again open times were not consistently altered (table 1). When statistical significance was not achieved, the trends in data were in generally the same direction as the significant effects, indicating increased activity with lower concentrations and shorter exposures followed by decreased activity with higher concentrations and longer exposures.

### (c) FKBP12 added to solubilized RyR2

In contrast to the one native RyR2 channel active in bilayers in 14 of 14 experiments, two to four solubilized RyR2 channels were active in bilayers in 13 of 21 experiments (figure 1*d*(ii)). With these multiple incorporations, channel activity was measured as fractional mean current (*I'*_F_), i.e. mean current divided by maximum current. *I'*_F_ reflects the average *P*_o_ of the two to four channels [28]. In analysis of single channels, relative *I'*_F_ (rel. *I'*_F_) is approximately equal to relative *P*_o_ (rel. *P*_o_), while with multiple channels rel. *I'*_F_ is approximately equal to the average *P*_o_ of the channels opening in the bilayer (addressed in electronic supplementary material, methods and figures S1–S4). To calculate an average open probability for data sets containing both single channel and multiple channel data, a term *P'* is defined (in which the individual *P*_o_ for single channel incorporations and individual *I'*_F_ values for multiple channel incorporations are combined to obtain an overall average for all data for each concentration of FKBP12. Notably, the effects of FKBP12 addition are very similar in average relative *P'* (for all bilayers) and average relative *P*_o_ for the subset of single channel incorporations (table 2 and figure 1*g*).

Because the activity of solubilized RyR2 was not affected by 1–10 nM FKBP12 in preliminary experiments (figure 1*d*(i)), more detailed experiments began with 200 nM FKBP12, where *P'* and *P*_o_ increased significantly at early and intermediate times then declined significantly at longer times (table 2 and figure 1*g*,*h*). The average activity declined significantly with longer exposures to 1 and 5 µM FKBP12. The activity did not increase significantly with briefer exposures to 1 and 5 µM FKBP12, presumably owing to an early onset of reduced activity seen in some channels (figure 1*d*(iii)). Gating parameters determined for the subset of single channel experiments revealed a significant shortening of closed durations when activity increased, or significant lengthening of closed durations when activity declined, with significant changes in event frequency (table 2 and figure 1*h*). Mean open times were not consistently altered throughout the experiments (table 2).

Overall, the results with P4 and solubilized RyR2 reveal time- and concentration-dependent transitions between a significant increase in activity above control levels (indicating activation) with lower FKBP12 concentrations and shorter exposure times and a significant decline in activity (indicating inhibition) to less than control levels with higher FKBP12 concentrations and longer exposures times.

### (d) The availability of FKBP12 binding sites on RyR2 channels

The availability of unoccupied FKBP12 binding sites on RyR2 channels was examined by incubating P4 vesicles with 10 µM GST-FKBP12, in excess of the estimated physiological levels in mouse myoctes of approximately 1 µM FKBP12, or 150 nM for FKBP12.6 [29]. Interestingly, when measured in mouse cardiac SR, however, the [FKBP12] was 48.6 pM per milligram of SR, with a 100-fold lower [FKBP12.6] of 0.48 pM per milligram of SR [6]. For the present experiment, the P4 vesicles were incubated both with and without GST-FKBP12 for 1 h at 37°C, before anti-RyR CoIP using the 34C antibody. RyR2 was detected with 34C antibody and both endogenous FKBP12/12.6 and exogenous GST-FKBP12 detected with the anti-FKBP12/12.6 H5 antibody (Methods and electronic supplementary material, methods).

The higher molecular mass of GST-FKBP12 was used to distinguish exogenous FKBP12 from endogenous FKBP (figure 2*a*(i)). To estimate the amount of additional FKBP-bound to RyR2 after exposure to GST-FKBP12, the normal dimer formation of GSTs [30,31] was factored into the calculations after confirming GST-FKBP12 also existed as a dimer using analytical centrifugation (figure 2*b*(i)). As a result of the dimerization, two GST-FKBP molecules would be detected with the binding of one FKBP to RyR2. This was accounted for before the construction of the graphs in figure 2*b*(ii), by assuming that only one FKBP12 molecule in the GST-FKBP12 dimer was associated with each FKBP binding site. Therefore, the density of the GST-FKBP12 band was halved to give the exogenous FKBP-bound to RyR2 (ex. FKBP12) before calculating the total FKBP associated with RyR2, i.e. (ex. + en. FKPB)/RyR2 ratio, and normalization to the endogenous (FKBP12 + FKBP12.6)/RyR2 ratio in control samples that were incubated under the same conditions but without GST-FKBP12 (legend to figure 2 and Methods). GST-cleaved FKBP12 did not dimerize at concentrations less than or equal to 10 µM, although it self-aggregated at concentrations greater than or equal to100 µM (figure 2*b*(ii)).

Substantial amounts of GST-FKBP12 were associated with RyR2 following incubation with GST-FKBP12 (figure 2*a*(i,ii)) and, at the same time, a substantial reduction in the amount of endogenous FKBP12/12.6 was apparent (figure 2*a*(i)). FKBP12 and FKBP12.6 both appeared to be reduced following exposure to GST-FKBP12, suggesting that both isoforms were replaced by exogenous FKBP12. A corresponding increase in FKBP12/12.6 in the supernatant (figure 2*a*(iii,iv)) verified the CoIP result and confirmed that GST-FKBP12 displaced much of the endogenous protein. GST-cleaved FKBP12 also produced a similar increase in total FKBP12 and demonstrated the GST itself did not bind substantially to RyR2 (figure 2*a*(v,vi)).

### (e) FKBP dissociation from RyR2 during processing

A significant loss of approximately 71% of FKBP12/12.6 between the homogenate and P4 vesicles was substantially reduced when the vesicles were processed with the FKBP-RyR-stabilizing drug S107 [26,27] (figure 2*c*(i,ii)). The loss of FKBP12/12.6 between P2 and P4 was also significantly reduced by S107 (figure 2*c*(iii,iv)). The combined results illustrate that the greatest loss of FKBP12 was between P2 and P4 and was largely prevented by S107 (figure 2*d*). Finally, addition of exogenous cleaved FKBP12 indicated that the homogenate itself was not saturated with FKBP (figure 2*e*). This raised the question of whether FKBP12 was lost from RyR2 during homogenization or whether RyR2 in myocytes was not saturated with FKBP12/12.6. Curiously, there was a trend towards a reduction in the exogenous FKBP12 binding to homogenate RyR2 in the presence of S107, which was not explored further.

### (f) Estimation of the fraction of FKBP binding sites occupied during SR processing

FKBP bound to RyR2 in the homogenate increased by 1.8-fold (without S107) or 1.5-fold (with S107) following saturation with exogenous FKBP12, indicating that approximately 2–3 (2.22–2.67) subunits contained FKBP before saturation (i.e. 4 × (1/1.8) = 2.22, or 4 × (1/1.5) = 2.67), assuming that FKBP was bound to all four subunits following saturation. The amount of endogenous FKBP bound to RyR2 in control P4 vesicles was estimated from three different experiments. Firstly, the loss of 72% of the homogenate FKBP in P4 (figure 2*c*(ii)) suggested a decline to less than 1 (0.62–0.75) of the RyR2 subunits containing FKBP. Secondly, a higher P4 occupation was predicted from the P4 saturation experiments with GST-FKBP12, which indicated a 2.5-fold increase in FKBP bound to RyR2 (figure 2*a***(**ii)), i.e. 1.6 FKBP-bound subunits in control P4 RyR2. Finally, the predicted amount of FKBP initially associated with P4 vesicles was a little higher when calculated from the 1.9-fold increase with cleaved FKBP12 binding to RyR2 (figure 2*a*(vi)). This indicated that 2.1 subunits contained endogenous FKBP prior to saturation. Together, the combined P4 data (ignoring the S107 data), i.e. (0.62 + 1.6 + 2.1)/3, suggest FKBP is bound to 1.44 subunits per P4 RyR2 tetramer. There would clearly be a distribution of channels with between zero and four FKBP-bound subunits per tetramer, but mainly one or two subunits containing FKBP as indicated in figure 3*a*, compared with an average 2.49 subunits in homogenate (mainly 2 containing FKBP in some tetramers and 3 in others).

The FKBP occupancy of solubilized RyR2 was not addressed in detail. However, the data in figure 1*c*(iii) suggest that the occupancy is approximately 5–10% of P4, so that at most 1.44 subunits in 10 tetramers would be FKBP-bound. In other words, most tetramers would not contain any FKBP, some would have FKBP bound to one subunit as suggested in figure 3*b*, and very occasionally two or more subunits might be occupied by FKBP.

## 4. Discussion

The unexpected biphasic changes in activity were consistently observed when FKBP12 was added to RyR2 channels. To explain these changes, we assume that there is one FKBP binding site on each RyR2 subunit, i.e. four sites per tetramer, and that FKBP12 and FKBP12.6 bind to this site [1,3,32]. We show that exogenous FKBP12 binds to RyR2 and exchanges with endogenous FKBP12/12.6. There was extensive dissociation of FKBP12/12.6 from RyR2 during SR vesicle processing, although measurable amounts remained associated with solubilized RyR2. We hypothesize that submaximal occupation of RyR2 subunits by FKBP12 in the channel tetramer can reduce the affinity of the unoccupied subunits for FKBP12.

### (a) Biphasic effects of FKBP12 on RyR2

The increase in RyR2 activity with low [FKBP12] and decrease at longer times or with higher [FKBP12] is consistent with the concept of higher affinity activation with partial FKBP occupation of the RyR2 subunit binding sites, followed by lower affinity inhibition as the number of occupied subunits increases. This is supported by clear transitions from activated to inhibited states in individual channels. The biphasic action is novel, although various reports in the literature find that FKBP12 can either inhibit, e.g. [14,15], or activate [13] RyR2 channels. Our previous observations are consistent FKBP being capable of both activation and inhibition. CLIC2, a normally inhibitory RyR-associated protein, dissociates FKBP12/12.6 from RyR1/RyR2 [8]. The CLIC2 H101Q mutant, associated with intellectual abnormalities, dissociates significantly more FKBP12/12.6 from RyR2 than WT CLIC2, but in contrast to WT CLIC2, it activates the channel. A biphasic effect of [FKBP12] has been suggested previously by others to explain the apparently contradictory evidence [7], but, in the absence of evidence at that time, was considered unlikely.

Our data also indicated that a higher [FKBP12] was required to produce similar effects in solubilized RyR2 compared with P4 channels. Although possibly a result of the greater loss of FKBP with solubilization, this is not consistent with a higher affinity for low occupancy binding. An equally plausible explanation among others is that the conformational consequences of removal of other associated regulatory proteins such as CSQ2, triadin and junctin [24,25] impacted on the nature of the FKBP binding site and altered the binding affinity.

### (b) Loss of FKBP during processing

In previous studies, either solubilization or treatment with high ionic strength, FK506 or rapamycin were used to strip FKBPs from RyRs, e.g. [13,33,34]. In our hands, none of those treatments is effective in stripping residual FKBP12 from RyR2 in P4 vesicles, although rapamycin partly reduces FKBP12.6 bound to RyR2 [8]. However, the present results suggest that most RyRs incorporated into bilayers from SR vesicles are at least partly stripped of endogenous FKBPs, with a progressive loss during SR vesicle processing and increased handling. At the same time, a population of FKBP12/12.6 remained associated with RyR2 even after solubilization, as previously reported [32], suggesting high affinity binding. It is conceivable that the FKBP remaining associated with RyR2 in P4 vesicles was bound with higher affinity and therefore resistant to dissociation with the various treatments [8].

## 5. A model for the effects of FKBP12 on RyR2 with negative cooperativity

The model in figure 3 is suggested to explain the time- and concentration-dependent changes in RyR2 activity following FKBP12 addition as well as the progressive loss of FKBP12/12.6 with processing, with some FKBP12/12.6 remaining associated with RyR2 even after solubilization. The model assumes that the number of subunits in the RyR2 tetramer that are occupied by FKBP12 increases with increasing [FKBP12] and with longer exposure times. Further assumptions are (i) that RyR2 affinity for FKBP12 is reduced as more subunits in the tetramer are occupied by FKBP12, (ii) that the highest affinity binding is to non-adjacent subunits, (iii) that binding to one subunit reduces the affinity for binding to neighbouring subunits, and (iv) that high affinity binding activates RyR2, while lower affinity binding inhibits the channel. The suggestion that a population of FKBP binds with higher affinity is consistent with the finding that some endogenous FKBP remained bound to RyR2 after solubilization and with reports of endogenous FKBP being visible in CryoEM of RyR1 purified without GST-FKBP precipitation [32]. A population of FKBP bound with lower affinity is suggested by the substantial amount of FKBP lost during processing to the P4 stage. The reduction in affinity as more FKBP12 binds to the RyR2 tetramer implies a negative cooperativity in this binding process.

In other words, we suggest that changes in affinity for FKBP binding can produce opposite effects on channel activity owing to the tetrameric structure of RyR2 channel protein because each of the four subunits contains a binding site for FKBP. The structure of the gating region of the channel is determined by molecular changes within each of the subunits and by interactions between adjacent subunits. The hypotheses are that:

(a) Residues in non-adjacent subunits are modified by FKBP binding in a manner that increases channel activity and reduces the affinity of their nearest neighbours for FKBP.
(b) FKBP binding with lower affinity to the adjacent subunits alters inter-subunit interactions in a manner that decreases channel activity.
(c) The probability of adjacent subunits being occupied by FKBP increases as more subunits are occupied by FKBP, with higher concentrations of FKBP and longer exposure times.

The model is consistent with preliminary MST experiments with rabbit skeletal RyR1 (figure 2*f*). RyR1, like RyR2, demonstrates time- and concentration-dependent changes in activity with FKBP12 addition and FKBP12 loss with processing (our unpublished observations). Prominent inflections in the normalized fluorescence at approximately 10 nM and approximately 1 µM RyR1 (figure 2*f*(i,ii)) indicate changes in affinity as the FKBP12/RyR1 ratio changes, because they were not seen when RyR1 was pre-saturated with FKBP12 (figure 2*g*(i,ii)). Although a more rigorous investigation is required, these preliminary data support the suggestion that there are changes in affinity with FKBP12 binding to more RyR subunits.

### (a) Molecular rearrangements could lead to changes in affinity for FKBP12

The assumptions in the model are supported by increasing numbers of elegant high-resolution CryoEM studies confirming a single FKBP binding site in each of the four RyR subunits. The investigations reveal in near-atomic detail the changes in RyR structure that underlie changes in channel gating due to structural rearrangement induced by ligand binding or by disease-associated mutations in RyRs [35,36]. FKBP interacts with the SPRY2 domain and α-solenoid 1 forming part of the corona [3], approximately 100 nm distant from the ion pore. Many ligands bind in this area and affect channel gating through a cascade of residue re-orientations along signalling pathways through the protein to the pore [37]. It is suggested that FKBP12 inhibits RyRs by clamping the SPRY2 and α-solenoid 1 domains and that reducing their mobility inhibits the channel [32,38]. Notably, the greatest mobility in RyRs is in the region of FKBP binding, which undergoes a downward rotation upon channel opening [7,38]. Disease-causing mutations in this area alter mobility throughout the protein and disrupt channel gating [36,39]. Thus far, the CryoEM studies have used FKBP-saturated RyRs, or RyR channels with FKBP largely absent, which differs from our conditions where RyR2 was less than saturated under control conditions, but still containing a substantial fraction of FKBP. There has not as yet been a structural comparison of RyR2 with different amounts of FKBP12 bound.

Our hypothesis is that conformational changes associated with FKBP binding to only one or two non-adjacent subunits differ from the changes associated with binding to adjacent subunits, which is obligatory when FKBP binds to three or four subunits. It is possible that the structural impact of FKBP binding to one or two non-adjacent subunits alters their orientation in such a way as to allow greater mobility within the tetramer. Greater mobility within the RyR due to ‘unzipping' of interdomain interactions has long been suggested to increase channel activity, while reduced-mobility ‘zipping' is suggested to reduce channel opening [32,35,36,38–43]. It is further conceivable that the structural changes within non-adjacent subunits increasing the overall tetramer mobility could reduce the affinity of unbound subunits for FKBP12. With sufficient increases in [FKBP12] and exposure time, the structural changes associated with FKBP binding to adjacent subunits may further alter inter-subunit interactions in such a manner as to reduce the overall of the mobility within the corona and thereby reduce channel activity.

### (b) Alternative hypotheses for a biphasic effect of FKBP12 on channel activity

Several alternative hypotheses exist. The three most likely are considered here, but the list is not exhaustive.

1. The biphasic effect of FKBP reflects FKBP binding to two separate sites with different affinities or accessibility. Binding to independent sites has been suggested to explain the biphasic effects of flecainide on RyR2 channel activity [44,45]. In contrast to FKBP12, the flecainide ligand binding sites on RyR2 have not been identified. The possibility of multiple flecainide binding sites is suggested by the promiscuity of the drug in binding to different proteins associated with cardiac excitation–contraction coupling [46]. Differential inhibitory actions of flecainide on RyR2 channel gating have similarly been attributed to independent interaction sites [45,47]. FKBP binds with high specificity to RyRs [48,49], and a single binding site on each of the RyR2 subunits has been clearly identified in the many high-resolution CryoEM studies, including those cited here. Therefore, it is unlikely that additional binding sites can explain the biphasic actions of FKBP12 on RyR2.
2. The biphasic effect of FKBP may reflect FKBP unbinding. Although a possibility, it is unlikely that either the increase in activity or the decrease in activity reflected FKBP12 unbinding because the maximum increases or decreases in activity were to levels significantly higher than, or significantly lower than, control levels, rather than a return to control levels, which might be expected with a simple unbinding.
3. FKBP interactions might alter binding of associated proteins to indirectly alter RyR activity. This could explain the result if the molecular changes associated with FKBP binding not only were transmitted to the pore but also influenced the conformation of binding sites for accessory proteins, causing their dissociation. For example, the luminal interaction between triadin, junctin and CSQ2 regulates channel activity [24,25] so that CSQ2 dissociates from the RyR2 complex, increasing channel activity, as in the CSQ2-null mouse [50]. It is conceivable that conformational changes associated with FKBP binding to RyR2 might be transmitted through the pore to luminal triadin/junctin binding sites on RyR2, thereby impacting on the CSQ2 binding properties of triadin and/or junctin. Alternatively, or in addition, the FKBP binding signal may be transmitted to the additional triadin and junctin binding sites on the cytoplasmic domain of the RyR that have been postulated for these transmembrane proteins [51,52], again altering their luminal association with CSQ2. However, the facts that solubilization removes triadin, junctin and CSQ2, and that similar overall effects of FKBP12 were seen in solubilized and native channels, provide a strong argument against alterations in associated protein interactions underlying the overall biphasic action of FKBP12.

### (c) How much FKBP is bound to RyR2 in the cell?

If the homogenate reflects the *in vivo* situation, then the channels in the cell are about 50% saturated with FKBP. Given the ease of dissociation, some FKBP may have been lost when the cells were disrupted, so that more RyR2 subunits may have contained FKBP in the cell. Previous studies using dog or mouse cardiac myocytes indicate that FKBP12.6 binding sites on RyR2 are 17 to 20% unoccupied [11,29]. However, it is difficult to compare amounts with the present study owing to species and FKBP isoform differences [6]. That some FKBP is easily dissociated is important physiologically and suggests a dynamic role for FKBP in responding to changes in cellular conditions that either enhance or reduce FKBP binding to RyR2, to modify RyR2 activity and diastolic Ca^2+^ leak. The *in vivo* consequences of FKBP12/12.6 regulation of RyR2 in cardiac myocytes are widely disputed. There is good evidence that FKBP12.6 association with RyR2 is decreased by PKA phosphorylation [53] and good evidence to the contrary [7]. *S*-nitrosylation is associated with FKBP12.6 dissociation from RyR2 [54]. There is strong evidence for [55] and against [16] Ca^2+^ leak from the SR in catecholaminergic polymorphic ventricular tachycardia (CPVT) being due to loss of KBP12.6. Given the report that FKBP12.6 does not alter RyR2 activity but prevents FKBP12 activation [13], FKBP12.6 might also prevent both the [FKBP12]-induced activation and inhibition reported here. Finally, the consequences of FKBP association/dissociation are likely to depend on the relative amounts of FKBP12 and 12.6 in the myocytes [6]. The results of *in vivo* exploration will depend critically on the experimental animal and the experimental protocol [7].

## 6. Conclusion

In conclusion, we describe significant changes in RyR2 channel activity that can be explained by the degree of saturation of RyR2 with FKBP12. There are significant changes in the amount of endogenous FKBP associated with RyR2 during processing of SR vesicles, which suggests that a substantial amount of FKBP is easily dissociated. Overall, the results suggest that FKBP12 might play a dynamic role in setting RyR2 channel activity and SR Ca^2+^ leak during diastole. As a result, channel activity might either increase or decrease depending on many factors, including the relative amounts of FKBP12/12.6 present in the cardiac myocytes, the degree of RyR2 phosphorylation and oxidation/nitrosylation, and of course the activity of other proteins that regulate Ca^2+^ concentrations in both the cytosol and the lumen of the SR [45], which both impact on RyR2 channel gating [45].

## Data Availability

Channel activity data were measured and analysed using Channel 2 (developed by P. W. Gage and M. Smith, John Curtin School of Medical Research) or Channel 3 (developed by N. W. Laver, University of Newcastle). For more details of this software please contact the corresponding author. The data are provided in the electronic supplementary material [56].

## References

[1] Zalk R, Clarke OB, Des Georges A, Grassucci RA, Reiken S, Mancia F, Hendrickson WA, Frank J, Marks AR. 2015 Structure of a mammalian ryanodine receptor. Nature **517**, 44-49. (10.1038/nature13950) 25470061 PMC4300236

[2] Wagenknecht T, Grassucci R, Berkowitz J, Wiederrecht GJ, Xin HB, Fleischer S. 1996 Cryoelectron microscopy resolves FK506-binding protein sites on the skeletal muscle ryanodine receptor. Biophys. J. **70**, 1709-1715. (10.1016/S0006-3495(96)79733-3) 8785329 PMC1225139

[3] Yan Z et al. 2015 Structure of the rabbit ryanodine receptor RyR1 at near-atomic resolution. Nature **517**, 50-55. (10.1038/nature14063) 25517095 PMC4338550

[4] Qi Y, Ogunbunmi EM, Freund EA, Timerman AP, Fleischer S. 1998 FK-binding protein is associated with the ryanodine receptor of skeletal muscle in vertebrate animals. J. Biol. Chem. **273**, 34 813-34 819. (10.1074/jbc.273.52.34813) 9857007

[5] Jeyakumar LH et al. 2001 FKBP binding characteristics of cardiac microsomes from diverse vertebrates. Biochem. Biophys. Res. Commun. **281**, 979-986. (10.1006/bbrc.2001.4444) 11237759

[6] Zissimopoulos S, Seifan S, Maxwell C, Williams AJ, Lai FA. 2012 Disparities in the association of the ryanodine receptor and the FK506-binding proteins in mammalian heart. J. Cell Sci. **125**, 1759-1769. (10.1242/jcs.098012) 22328519

[7] Gonano LA, Jones PP. 2017 FK506-binding proteins 12 and 12.6 (FKBPs) as regulators of cardiac ryanodine receptors: insights from new functional and structural knowledge. Channels **11**, 415-425. (10.1080/19336950.2017.1344799) 28636428 PMC5626368

[8] Richardson SJ, Steele GA, Gallant EM, Lam A, Schwartz CE, Board PG, Casarotto MG, Beard NA, Dulhunty AF. 2017 Association of FK506 binding proteins with RyR channels: effect of CLIC2 binding on sub-conductance opening and FKBP binding. J. Cell Sci. **130**, 3588-3600. (10.1242/jcs.204461) 28851804

[9] Walweel K, Molenaar P, Imtiaz MS, Denniss A, Dos Remedios C, van Helden DF, Dulhunty AF, Laver DR, Beard NA. 2017 Ryanodine receptor modification and regulation by intracellular Ca^2+^ and Mg^2+^ in healthy and failing human hearts. J. Mol. Cell. Cardiol. **104**, 53-62. (10.1016/j.yjmcc.2017.01.016) 28131631

[10] Barg S, Copello JA, Fleischer S. 1997 Different interactions of cardiac and skeletal muscle ryanodine receptors with FK-506 binding protein isoforms. Am. J. Physiol. **272**, C1726-C1733. (10.1152/ajpcell.1997.272.5.C1726) 9176165

[11] Timerman AP, Onoue H, Xin H-B, Barg S, Copello J, Wiederrecht G, Fleischer S. 1996 Selective binding of FKBP12.6 by the cardiac ryanodine receptor. J. Biol. Chem. **271**, 20 385-20 391. (10.1074/jbc.271.34.20385) 8702774

[12] Xiao J et al. 2007 Removal of FKBP12.6 does not alter the conductance and activation of the cardiac ryanodine receptor or the susceptibility to stress-induced ventricular arrhythmias. J. Biol. Chem. **282**, 34 828-34 838. (10.1074/jbc.M707423200) PMC2760432 17921453

[13] Galfré E, Pitt SJ, Venturi E, Sitsapesan M, Zaccai NR, Tsaneva-Atanasova K, O'Neill S, Sitsapesan R. 2012 FKBP12 activates the cardiac ryanodine receptor Ca^2+^-release channel and is antagonised by FKBP12.6. PLoS ONE **7**, e31956. (10.1371/journal.pone.0031956) 22363773 PMC3283708

[14] Marx SO, Reiken S, Hisamatsu Y, Jayaraman T, Burkhoff D, Rosemblit N, Marks AR. 2000 PKA phosphorylation dissociates FKBP12.6 from the calcium release channel (ryanodine receptor): defective regulation in failing hearts. Cell **101**, 365-376. (10.1016/S0092-8674(00)80847-8) 10830164

[15] Wehrens XH, Lehnart SE, Marks AR. 2005 Intracellular calcium release and cardiac disease. Annu. Rev. Physiol. **67**, 69-98. (10.1146/annurev.physiol.67.040403.114521) 15709953

[16] Zhang JZ, Waddell HM, Wu E, Dholakia J, Okolo CA, McLay JC, Jones PP. 2016 FKBPs facilitate the termination of spontaneous Ca^2+^ release in wild-type RyR2 but not CPVT mutant RyR2. Biochem. J. **473**, 2049-2060. (10.1042/BCJ20160389) 27154203

[17] Chamberlain BK, Fleischer S. 1988 Isolation of canine cardiac sarcoplasmic reticulum. Methods Enzymol. **157**, 91-99. (10.1016/0076-6879(88)57071-4) 3231096

[18] Laver DR, Roden LD, Ahern GP, Eager KR, Junankar PR, Dulhunty AF. 1995 Cytoplasmic Ca^2+^ inhibits the ryanodine receptor from cardiac muscle. J. Membr. Biol. **147**, 7-22. (10.1007/BF00235394) 8531200

[19] Salvage SC, Gallant EM, Beard NA, Ahmad S, Valli H, Fraser JA, Huang CL-H, Dulhunty AF. 2019 Ion channel gating in cardiac ryanodine receptors from the arrhythmic RyR2-P2328S mouse. J. Cell Sci. **132**, jcs229039. (10.1242/jcs.229039) 31028179 PMC6550012

[20] Copello JA, Barg S, Onoue H, Fleischer S. 1997 Heterogeneity of Ca^2+^ gating of skeletal muscle and cardiac ryanodine receptors. Biophys. J. **73**, 141-156. (10.1016/S0006-3495(97)78055-X) 9199779 PMC1180916

[21] Mackrill JJ, O'driscoll S, Lai FA, Mccarthy TV. 2001 Analysis of type 1 ryanodine receptor-12kDa FK506-binding protein interaction. Biochem. Biophys. Res. Commun. **285**, 52-57. (10.1006/bbrc.2001.5125) 11437371

[22] Zhao H, Piszczek G, Schuck P. 2015 SEDPHAT–a platform for global ITC analysis and global multi-method analysis of molecular interactions. Methods **76**, 137-148. (10.1016/j.ymeth.2014.11.012) 25477226 PMC4380758

[23] Jerabek-Willemsen M, Wienken CJ, Braun D, Baaske P, Duhr S. 2011 Molecular interaction studies using microscale thermophoresis. Assay Drug Dev. Technol. **9**, 342-353. (10.1089/adt.2011.0380) 21812660 PMC3148787

[24] Györke I, Hester N, Jones LR, Györke S. 2004 The role of calsequestrin, triadin, and junctin in conferring cardiac ryanodine receptor responsiveness to luminal calcium. Biophys. J. **86**, 2121-2128. (10.1016/S0006-3495(04)74271-X) 15041652 PMC1304063

[25] Wei L, Gallant EM, Dulhunty AF, Beard NA. 2009 Junctin and triadin each activate skeletal ryanodine receptors but junctin alone mediates functional interactions with calsequestrin. Int. J. Biochem. Cell Biol. **41**, 2214-2224. (10.1016/j.biocel.2009.04.017) 19398037 PMC2777989

[26] Shan J, Xie W, Betzenhauser M, Reiken S, Chen B-X, Wronska A, Marks AR. 2012 Calcium leak through ryanodine receptors leads to atrial fibrillation in 3 mouse models of catecholaminergic polymorphic ventricular tachycardia. Circ. Res. **111**, 708-717. (10.1161/CIRCRESAHA.112.273342) 22828895 PMC3734386

[27] Xie W, Santulli G, Guo X, Gao M, Chen B-X, Marks AR. 2013 Imaging atrial arrhythmic intracellular calcium in intact heart. J. Mol. Cell. Cardiol. **64**, 120-123. (10.1016/j.yjmcc.2013.09.003) 24041536 PMC4387875

[28] Gallant EM, Curtis S, Pace SM, Dulhunty AF. 2001 Arg^615^Cys substitution in pig skeletal ryanodine receptors increases activation of single channels by a segment of the skeletal DHPR II-III loop. Biophys. J. **80**, 1769-1782. (10.1016/S0006-3495(01)76147-4) 11259290 PMC1301366

[29] Guo T, Cornea RL, Huke S, Camors E, Yang Y, Picht E, Fruen BR, Bers DM. 2010 Kinetics of FKBP12.6 binding to ryanodine receptors in permeabilized cardiac myocytes and effects on Ca sparks. Circ. Res. **106**, 1743-1752. (10.1161/CIRCRESAHA.110.219816) 20431056 PMC2895429

[30] Ji X, Zhang P, Armstrong RN, Gilliland GL. 1992 The three-dimensional structure of a glutathione *S*-transferase from the Mu gene class. Structural analysis of the binary complex of isoenzyme 3–3 and glutathione at 2.2-Å resolution. Biochemistry **31**, 10 169-10 184. (10.1021/bi00157a004) 1420139

[31] Parker MW, Lo Bello M, Federici G. 1990 Crystallization of glutathione *S-*transferase from human placenta. J. Mol. Biol. **213**, 221-222. (10.1016/S0022-2836(05)80183-4) 2342105

[32] Efremov RG, Leitner A, Aebersold R, Raunser S. 2015 Architecture and conformational switch mechanism of the ryanodine receptor. Nature **517**, 39-43. (10.1038/nature13916) 25470059

[33] Ahern GP, Junankar PR, Dulhunty AF. 1997 Subconductance states in single-channel activity of skeletal muscle ryanodine receptors after removal of FKBP12. Biophys. J. **72**, 146-162. (10.1016/S0006-3495(97)78654-5) 8994600 PMC1184304

[34] Venturi E, Galfré E, O'Brien F, Pitt SJ, Bellamy S, Sessions RB, Sitsapesan R. 2014 FKBP12.6 activates RyR1: investigating the amino acid residues critical for channel modulation. Biophys. J. **106**, 824-833. (10.1016/j.bpj.2013.12.041) 24559985 PMC3945099

[35] Chi X, Gong D, Ren K, Zhou G, Huang G, Lei J, Zhou Q, Yan N. 2019 Molecular basis for allosteric regulation of the type 2 ryanodine receptor channel gating by key modulators. Proc. Natl Acad. Sci. USA **116**, 25 575-25 582. (10.1073/pnas.1914451116) PMC6926060 31792195

[36] Iyer KA, Hu Y, Nayak AR, Kurebayashi N, Murayama T, Samsó M. 2020 Structural mechanism of two gain-of-function cardiac and skeletal RyR mutations at an equivalent site by cryo-EM. Sci. Adv. **6**, eabb2964. (10.1126/sciadv.abb2964) 32832689 PMC7439390

[37] Dulhunty AF. 2022 Molecular changes in the cardiac RyR2 with catecholaminergic polymorphic ventricular tachycardia (CPVT). Front. Physiol. **13**, 830367. (10.3389/fphys.2022.830367) 35222090 PMC8867003

[38] Steele TWE, Samso M. 2019 The FKBP12 subunit modifies the long-range allosterism of the ryanodine receptor. J. Struct. Biol. **205**, 180-188. (10.1016/j.jsb.2018.12.007) 30641143 PMC6494473

[39] Woll KA, Haji-Ghassemi O, Van Petegem F. 2021 Pathological conformations of disease mutant ryanodine receptors revealed by cryo-EM. Nat. Commun. **12**, 807. (10.1038/s41467-021-21141-3) 33547325 PMC7864917

[40] Bannister ML, Hamada T, Murayama T, Harvey PJ, Casarotto MG, Dulhunty AF, Ikemoto N. 2007 Malignant hyperthermia mutation sites in the Leu2442-Pro2477 (DP4) region of RyR1 (ryanodine receptor 1) are clustered in a structurally and functionally definable area. Biochem. J. **401**, 333-339. (10.1042/BJ20060902) 16958617 PMC1698659

[41] Lamb GD, Posterino GS, Yamamoto T, Ikemoto N. 2001 Effects of a domain peptide of the ryanodine receptor on Ca^2+^ release in skinned skeletal muscle fibers. Am. J. Physiol. Cell Physiol. **281**, C207-C214. (10.1152/ajpcell.2001.281.1.C207) 11401843

[42] Shtifman A, Ward CW, Yamamoto T, Wang J, Olbinski B, Valdivia HH, Ikemoto N, Schneider MF. 2002 Interdomain interactions within ryanodine receptors regulate Ca^2+^ spark frequency in skeletal muscle. J. Gen. Physiol. **119**, 15-32. (10.1085/jgp.119.1.15) 11773235 PMC2233852

[43] Yamamoto T, El-Hayek R, Ikemoto N. 2000 Postulated role of interdomain interaction within the ryanodine receptor in Ca^2+^ channel regulation. J. Biol. Chem. **275**, 11 618-11 625. (10.1074/jbc.275.16.11618) 10766778

[44] Salvage SC, Gallant EM, Fraser JA, Huang CL-H, Dulhunty AF. 2021 Flecainide paradoxically activates cardiac ryanodine receptor channels under low activity conditions: a potential pro-arrhythmic action. Cells **10**, 2101. (10.3390/cells10082101) 34440870 PMC8394964

[45] Salvage SC, Huang CL-H, Fraser JA, Dulhunty AF. 2022 How does flecainide impact RyR2 channel function? J. Gen. Physiol. **154**, e202213089. (10.1085/jgp.202213089) 35713932 PMC9208819

[46] Salvage SC, Chandrasekharan KH, Jeevaratnam K, Dulhunty AF, Thompson AJ, Jackson AP, Huang CL-H. 2018 Multiple targets for flecainide action: implications for cardiac arrhythmogenesis. Br. J. Pharmacol. **175**, 1260-1278. (10.1111/bph.13807) 28369767 PMC5866987

[47] Mehra D, Imtiaz MS, van Helden DF, Knollmann BC, Laver DR. 2014 Multiple modes of ryanodine receptor 2 inhibition by flecainide. Mol. Pharmacol. **86**, 696-706. (10.1124/mol.114.094623) 25274603 PMC4244595

[48] Jayaraman T, Brillantes AM, Timerman AP, Fleischer S, Erdjument-Bromage H, Tempst P, Marks AR. 1992 FK506 binding protein associated with the calcium release channel (ryanodine receptor). J. Biol. Chem. **267**, 9474-9477. (10.1016/S0021-9258(19)50114-4) 1374404

[49] Timerman AP, Jayaraman T, Wiederrecht G, Onoue H, Marks AR, Fleischer S. 1994 The ryanodine receptor from canine heart sarcoplasmic reticulum is associated with a novel FK-506 binding protein. Biochem. Biophys. Res. Commun. **198**, 701-706. (10.1006/bbrc.1994.1101) 8297381

[50] Knollmann BC et al. 2006 *Casq2* deletion causes sarcoplasmic reticulum volume increase, premature Ca^2+^ release, and catecholaminergic polymorphic ventricular tachycardia. J. Clin. Invest. **116**, 2510-2520. (10.1172/JCI29128) 16932808 PMC1551934

[51] Groh S, Marty I, Ottolia M, Prestipino G, Chapel A, Villaz M, Ronjat M. 1999 Functional interaction of the cytoplasmic domain of triadin with the skeletal ryanodine receptor. J. Biol. Chem. **274**, 12 278-12 283. (10.1074/jbc.274.18.12278) 10212196

[52] Li L et al. 2015 A new cytoplasmic interaction between junctin and ryanodine receptor Ca^2+^ release channels. J. Cell Sci. **128**, 951-963. (10.1242/jcs.160689) 25609705 PMC4342579

[53] Wehrens XHT, Lehnart SE, Reiken S, Vest JA, Wronska A, Marks AR. 2006 Ryanodine receptor/calcium release channel PKA phosphorylation: a critical mediator of heart failure progression. Proc. Natl Acad. Sci. USA **103**, 511-518. (10.1073/pnas.0510113103) 16407108 PMC1334677

[54] Fauconnier J, Thireau J, Reiken S, Cassan C, Richard S, Matecki S, Marks AR, Lacampagne A. 2010 Leaky RyR2 trigger ventricular arrhythmias in Duchenne muscular dystrophy. Proc. Natl Acad. Sci. USA **107**, 1559-1564. (10.1073/pnas.0908540107) 20080623 PMC2824377

[55] Lehnart SE, Wehrens XH, Kushnir A, Marks AR. 2004 Cardiac ryanodine receptor function and regulation in heart disease. Ann. NY Acad. Sci. **1015**, 144-159. (10.1196/annals.1302.012) 15201156

[56] Richardson SJ, Thekkedam CG, Casarotto MG, Beard NA, Dulhunty AF. 2023 FKBP12 binds to the cardiac ryanodine receptor with negative cooperativity: implications for heart muscle physiology in health and disease. Figshare. (10.6084/m9.figshare.c.6498533) PMC10150220 37122219

